# *Campylobacter jejuni *isolates in Finnish patients differ according to the origin of infection

**DOI:** 10.1186/1757-4749-2-22

**Published:** 2010-12-20

**Authors:** Benjamin Feodoroff, Patrik Ellström, Heidi Hyytiäinen, Seppo Sarna, Marja-Liisa Hänninen, Hilpi Rautelin

**Affiliations:** 1 Department of Bacteriology and Immunology, Haartman Institute, University of Helsinki, Haartmaninkatu 3, PO Box 21, FIN-00014, Helsinki, Finland; 2 Department of Medical Sciences, University of Uppsala, S-75185 Uppsala, Sweden; 3 Department of Food and Environmental Hygiene, University of Helsinki, PO Box 66, FIN-00014, Helsinki, Finland; 4 Department of Public Health, University of Helsinki, Mannerheimintie 172, PO Box 41, FIN-00014, Helsinki, Finland; 5 Helsinki University Central Hospital Laboratory, Helsinki, Finland

## Abstract

**Background:**

*Campylobacter jejuni *is a significant cause of bacterial enteritis worldwide. Very little is known about the pathogenicity mechanisms and virulence factors of this important enteropathogen. *C. jejuni *isolates from 166 Finnish patients, collected from July to December in 2006, were studied for the presence of putative virulence factors and susceptibility to antimicrobials. Isolates were tested for production of γ-glutamyltransferase (GGT) as well as the presence of genes *ceuE*, *cgtB*, *ciaB*, *cj0486*, *pldA*, *virB11*, *wlaN*, and the gene cluster *cdtABC*. Bacterial characteristics were compared to information on foreign travel history as well as information on the course and the symptoms of disease obtained from questionnaires returned by patients.

**Results:**

Except for one domestic isolate, antimicrobial resistance was only detected in isolates of foreign origin. Univariate analyses showed association between bloody stools and both GGT production (p = 0.025) and the presence of *cgtB *(p = 0.034). Multivariate analysis verified that GGT production was more prevalent in domestic isolates (p < 0.0001), while the genes *cj0486 *(p < 0.0001) and *ceuE *(p < 0.0001) were associated with *C. jejuni *isolates of foreign origin.

**Conclusions:**

The results indicate that imported and domestic *C. jejuni *isolates differ significantly in several aspects from each other.

## Background

*Campylobacter jejuni *is a leading cause of bacterial enteritis in developed countries and the most commonly reported zoonosis in the European Union [1]. *C. jejuni *colonizes the gastrointestinal tract of many animals including poultry and wild birds, cattle, pigs, cat and dog. Eating undercooked poultry has been shown to be a risk factor for campylobacteriosis also in Finland [2], however, poultry is colonized with *Campylobacter *to a significantly lower extent than in many other countries [3]. Epidemiological studies using serotyping and genotyping methods have revealed a high diversity among *C. jejuni *from different sources in Finland and the risk factors for human *Campylobacter *infection may vary according to the geographical area and even with age [4,5].

Although the genomes of several *C. jejuni *strains have been sequenced [6-8], very little is still known about the pathogenicity mechanisms and the virulence factors of this common enteropathogen [9]. The acute *Campylobacter *infection is often self-limited but in severe cases antimicrobial and hospital treatment may be needed. The reasons why certain patients develop a more serious acute infection or late sequelae of the disease are not understood.

Several studies have searched for the presence of putative virulence factors among *Campylobacter *isolates of human and animal origin but only few studies have been able to show an association between certain bacterial factors and the outcome of human *Campylobacter *infection. Genes studied have usually included those suggested to have a role in adhesion, colonization, invasion and toxin production. The plasmid-associated gene *virB11 *[10], as well as the genes *ciaB *(*Campylobacter *invasive antigen B) [11,12], and *cj0486 *encoding a putative sugar transporter [13] have been suggested to be involved in invasiveness. In addition, *pldA *encoding outer membrane phospholipase A [14], and *ceuE *encoding enterochelin uptake binding protein [15] have been studied. The genes *cgtB *[16] and *wlaN *[17] are involved in the biosynthesis of lipooligosaccharide (LOS), which may show ganglioside-mimicking structures important for the triggering of Guillain-Barré syndrome, an acute peripheral polyneuropathy, after *C. jejuni *infection [18]. Cytolethal distending toxin (CDT, encoded by the gene cluster *cdtABC*) has been present in most of the tested isolates [19] but its role in the outcome of the disease still remains uncertain. Likewise, a recent report showed γ-glutamyl transpeptidase (GGT, encoded by the gene *ggt*) to have a role in the persistent colonization of the avian gut [20], but its importance in the course of human campylobacteriosis is not known.

We have recently demonstrated that *C. jejuni *isolates of domestic origin and those highly susceptible for ciprofloxacin were associated with a more severe form of enteritis characterized by bloody stools [21]. Our aim in the present study was to reveal other bacterial factors that may affect the outcome of the *C. jejuni *infection in a well-characterized clinical material including both infections acquired abroad or in Finland. For that purpose, we analyzed 166 clinical isolates of *C. jejuni *for the production of GGT and the presence of the genes *virB11*, *ciaB*, *cgtB*, *wlaN*, *cj0486*, *ceuE*, *pldA *and *cdtABC*.

## Methods

### Patients and *Campylobacter *isolates

A total of 166 patients with sporadic stool culture-verified *C. jejuni *infection (no other bacterial enteropathogens detected) were included in the present study. Their stool samples were collected from July 1 to December 31, 2006, and they had returned questionnaires including questions concerning travelling abroad within two weeks before the onset of symptoms, the clinical course of the illness and antimicrobial therapy. The patients belonged to a group of 192 persons (originally also including *C. coli *positive patients), some results of which have earlier been presented [21]. All the isolates were hippurate positive and were stored at -70°C before analyzed.

### Susceptibility testing

The minimal inhibitory concentration (MIC) values of ciprofloxacin (Bayer, Leverkusen, Germany), doxycycline (Orion Pharma, Espoo, Finland) and erythromycin (Amdipharm Ltd, Dublin, Ireland) were determined by an agar dilution method according to the CLSI guidelines [22]. Mueller-Hinton agar (Oxoid, Basingstoke, UK) plates supplemented with defibrinated sheep blood (5%) and the control strain *C. jejuni *ATCC 33560 were used. The susceptibility of the isolates was interpreted according to CLSI [23]. The results of susceptibility of the isolates to ciprofloxacin have been published earlier [21].

### PFGE typing

PFGE analysis was performed for 75 (all 40 domestic and all 35 travel-associated isolates collected in July, as the prevalence of cases per month was the highest during this month) isolates as described earlier [3,24]. Isolate was considered to represent a new type when PFGE *KpnI *profile differed by at least one band.

### γ-Glutamyl transpeptidase activity

In the first phase we studied the presence or absence of *ggt *gene as described in our previous study [25]. All isolates positive for the gene were further analyzed for the production of GGT. Qualitative detection of GGT activity was achieved as described previously for *Helicobacter pylori *[26]. Briefly, approximately 10^9 ^bacteria were suspended in a reagent containing 50 mM Tris (pH 8.25), 1.5 mM L-γ-glutamyl-carboxyl-3 nitro-4 anilide and 50 mM

glycylglycine. The mixture was incubated for 1 h at 37°C. Cleavage of the substrate by γ-glutamyl transpeptidase turned the mixture yellow in color.

### PCR detection of other putative *C. jejuni *virulence factors

DNA was extracted from *C. jejuni *isolates using the following protocols. Bacteria (10^8 ^CFU) were harvested from blood agar plates (Columbia agar II containing 8% vol/vol whole horse blood), dissolved in 500 μl of ddH_2_O and incubated in a boiling water bath for 10 min. Cell debris was removed by centrifugation at 18 000 g for 2 min. For some isolates DNA was extracted using either a method utilizing guanidium thiocyanate [27], DNeasy Blood & Tissue kit (Qiagen) or Wizard Genomic DNA Purification Kit (Promega, UK) according to the manufacturer's instructions. Successful extraction of template *C. jejuni *DNA from each isolate was confirmed by PCR amplification of the house keeping gene *glyA*. The presence of the gene *glyA *and the putative virulence factors *virB11*, *ciaB*, *cgtB*, *wlaN*, *pldA*, *ceuE*, *Cj0486 *as well as the *cdtABC *operon were determined by PCR using the primers listed in Table 1. The reaction mixture was prepared in 1× AmpliTaq Gold 360 buffer with 1.25U of AmpliTaq Gold 360 polymerase (Applied Biosystems, USA), 200 μM dNTP (Fermentas, Germany), 0.2 μM of each primer (Eurogentec, Ougrée, Belgium) and 5 μl of template DNA in a total volume of 25 μl.

**Table 1:** Primer sequences, annealing temperatures and PCR product sizes for the putative virulence factors studied

Gene	Primers	Sequence (5'-3')	Annealing temp (°C)	Product (bp)	Reference
*gly*A	Gly-Fw Gly-rev	GAGTTAGAGCGTCAATGTGAAGG AAACCTCTGGCAGTAAGGGC	53-51	1052	[36]

*virB11*	VirB-232 VirB-701	TCTTGTGAGTTGCCTTACCCCTTTT CCTGCGTGTCCTGTGTTATTTACCC	53	494	[35]

*ceu*E	CeuE405F CeuE405R	GATAAAGTCGTTGGCGTTCC GCGAGATTGGAGGACCAAAGG	60	405	*

*cia*B	ciaB355F ciaB355R	CAGAAGGAGAAATTTGTGAGC ATATCCCATTCTAATGCCACC	58	355	*

*pld*A	pldA-84fwd pld-981rev	AAGCTTATGCGTTTTT TATAAGGCTTTCTCCA	45	913	[35]

*Cj0486*	Cj0486fwd Cj0486rev	GATAGAGCATTAAATTGGGATG CCTATAAAGCCATACCAAGCC	58	1263	[13]

*wla*N	wlaN-DL 39 Cj1139cF	TTAAGAGCAAGATATGAAGGTG TGCTGGGTATACAAAGGTTGTG	60	434	[17,37]

*cgt*B	wlaN-DL 39 cgtBrev	TTAAGAGCAAGATATGAAGGTG GCACATAGAGAACGCTACAA	56	563	[17]

*cdtABC*	LYA-F MII-R	CTTTATGCATGTTCTTCTAAATTT GTTAAAGGTGGGGTTATAATCATT	55	2111	[38]

*Personal communication: Rafal Gierczyński, National Institute of Public Health, Warsaw, Poland.

The PCR reactions started with a denaturation step at 95°C for 10 min. The cycling conditions were 25 cycles of 95°C for 30 s, annealing temperatures (Table 1) for 30 s and 72°C for 60 s (120 s for *cdtABC*). For *virB11 *and *glyA *a touch down protocol was run with 5 cycles at 53°C, 5 cycles at 52°C and 15 cycles at 51°C. The reactions ended with an additional extension step at 72°C for 7 min. DNA extracted from *C. jejuni *NCTC 11168 was used as a positive control for the genes *cdtABC*, *wlaN *and *Cj0486 *and DNA from *C. jejuni *81176 served as a positive control for all other virulence genes. A PCR reaction without added template was used as a negative control.

### Statistical analyses

Statistical analyses were performed with GraphPad Prism version 4.03 (GraphPad Software, San Diego, CA, USA) and PASW Statistics 18 (SPSS for Windows, Rel. 18.0.2. 2010. SPSS Inc, Chicago, IL, USA). The χ^2 ^test and Fisher's exact test were used for comparison of categorical variables. Multivariate analyses were performed with stepwise binary logistic regression models. All tests were two-sided, and a p-value < 0.05 was considered to be significant.

The study was approved by the Ethics Committee of the Hospital District of Helsinki and Uusimaa.

## Results

Of the 166 cases of *C. jejuni *infection in the study 126 were acquired abroad and 40 were acquired in Finland. Resistance to erythromycin (4 isolates) and resistance to doxycycline (59 isolates) were only detected in isolates of foreign origin. All except one of the isolates of domestic origin were susceptible for ciprofloxacin whereas 83/126 (66%) of isolates of foreign origin were resistant to ciprofloxacin, as published earlier [21]. Prevalence of the putative virulence markers among the isolates according to MICs to ciprofloxacin and doxycycline, respectively, is presented in Table 2. GGT-production was associated with susceptibility to ciprofloxacin and doxycycline in the univariate analysis. On the other hand, both ciprofloxacin- and doxycycline-resistant isolates were more likely than the susceptible ones to harbor the genes *cj0486 *and *ceuE *(Table 2).

**Table 2:** Contingency table results for antimicrobial susceptibility and putative virulence markers among 166 *C. jejuni* isolates

MIC values	Putative virulence factor								
	GGT-production	*vir*B11	*cia*B	*cgt*B	*wla*N	*cj*0486	*ceu*E	*pld*A	*cdtABC*
Ciprofloxacin MIC ≤ 1 (82/166) *	20/82 (p = 0.001) §	1/82	81/82	16/82	17/82	31/82	62/82	52/82	69/82
Ciprofloxacin MIC ≥ 4 (84/166) †	5/84	3/84	83/84	15/84	22/84	50/84 (p = 0.0051) ¶	80/84 (p = 0.0003) ¶	49/84	62/84
									
Doxycycline MIC ≤ 2 (102/166) *	22/102 (p = 0.0032) §	1/102	101/102	22/102	20/102	40/102	82/102	59/102	82/102
Doxycycline MIC ≥ 4 (64/166) ‡	3/64	3/64	63/64	9/64	19/64	41/64 (p = 0.0018) #	60/64 (p < 0.0001) #	42/64	49/64
									

No. positive isolates (%)	25/166 (15%)	4/166 (2.4%)	164/166 (99%)	31/166 (19%)	39/166 (23%)	81/166 (49%)	142/166 (86%)	101/166 (61%)	131/166 (79%)

*interpreted as susceptible, †interpreted as resistant, ‡interpreted as resistant or intermediate (5 isolates with MIC 4 mg/L), §associated with susceptible isolates, ¶associated with resistant isolates, #associated with resistant or intermediate isolates

Contingency tables were also used to assess whether the presence of putative virulence factors correlated with clinical data. In the univariate analysis (Table 3), GGT production and the presence of the gene *cgtB *were associated with bloody stools. In addition, isolates lacking the *ceuE *gene were associated with hospitalization, as 8/24 (33%) of the patients infected with *ceuE*-negative isolates were hospitalized, compared to 21/139 (15%) of those with *ceuE*-positive isolates (p = 0.031). GGT production was strongly associated with infections acquired in Finland as compared to infections from abroad. On the other hand, the genes *cj0486 *and *ceuE *were markedly more common among isolates of foreign origin. Of all the domestic isolates, 34/40 (85%) showed at least one of the following characteristics; GGT-production, lack of *cj0486 *or absence of *ceuE *whereas the number of foreign isolates was 60 (48%), respectively (p < 0.0001). A total of 69/126 (55%) of the imported isolates, but only 7/40 (18%) of the domestic isolates were positive for both *cj0486 *and *ceuE *(p < 0.0001).The significant findings in the univariate model were controlled with a multivariate analysis to assess whether some of the variables were independently associated with each other. GGT-production was independently associated with domestic infections, and the genes *cj0486 *and *ceuE *were independently associated with imported infections, respectively (Table 4). Although bloody diarrhea was significantly associated with both GGT-production and the presence of *cgtB *in the univariate analysis, this factor could not be further analyzed with a multivariate analysis as the proportion of missing data in the questionnaires regarding this specific finding was too high (28%).

**Table 3:** Characteristics of 166 patients and putative virulence factors present in the respective *C. jejuni* isolates

Clinical characteristics	Putative virulence factor								
	GGT-production	*vir*B11	*cia*B	*cgt*B	*wla*N	*cj*0486	*Ceu*E	*pld*A	*cdt*ABC
Female sex (99/166)	17/99	3/99	98/99	19/99	23/99	52/99	85/99	62/99	78/99
Underlying disease (38/162)	9/38	3/38	37/38	10/38	9/38	18/38	32/38	23/38	31/38
Domestic infection (40/166)	16/40 (p < 0.0001) *	1/40	40	9/40	8/40	7/40	26/40	25/40	34/40
Infection from abroad 126/166)	9/126	3/126	124/126	22/126	31/126	74/126 (p < 0.0001) †	116/126 (p < 0.0001) †	76/126	97/126
Vomiting (41/156)	8/41	1/41	40/41	8/41	9/41	17/41	34/41	25/41	32/41
Fever (136/156)	20/136	4/136	134/136	27/136	30/136	66/136	115/136	82/136	104/136
Bloody stools (21/119)	6/21 (p = 0.025)	1/21	21	8/21 (p = 0.034)	3/21	9/21	17/21	16/21	18/21
Long-lasting (≥ 10 d) diarrhea (42/161)	6/42	1/42	42	12/42 (p = 0.051)	7/42	18/42	35/42	24/42	31/42
Hospitalization (29/163)	3/29	1/29	29	7/29	8/29	15/29	21/29 (p = 0.031) ‡	19/29	23/29

No. positive isolates (%)	25/166 (15%)	4/166 (2.4%)	164/166 (99%)	31/166 (19%)	39/166 (23%)	81/166 (49%)	142/166 (86%)	101/166 (61%)	131/166 (79%)

*associated with domestic infection, †associated with infection from abroad, ‡the absence of *ceu*E associated with hospitalization

**Table 4:** Multivariate analysis showing independent association between origin of infection and certain *C. jejuni* markers

Virulence marker	OR	95% Confidence interval	p-value
GGT*	8.67	3.43-21.91	< 0.0001

*cj*0486†	6.71	2.75-16.39	< 0.0001

*ceu*E†	6.71	2.67-16.95	< 0.0001

*associated with domestic infection, †associated with imported infection

PFGE analysis with *KpnI *digested samples revealed that among the 40 domestic isolates, a total of 32 PFGE types were identified indicating a high diversity. Furthermore, all the 35 isolates of foreign origin analyzed had different PFGE profiles, which did not overlap with those of domestic isolates. Thus, the isolates were highly diverse and did not seem to have a common source.

## Discussion

We recently showed that bloody stools were more common among patients infected with *C. jejuni *isolates of domestic origin and those highly susceptible for ciprofloxacin [21]. *C. jejuni *isolates of Finnish origin have even earlier been shown to differ significantly from those of foreign origin as being almost exclusively susceptible for ciprofloxacin [28,29]. In the present study, we further analyzed possible differences between *C. jejuni *isolates acquired in Finland and those from abroad and screened for the presence of certain putative virulence markers. Significant differences were detected as GGT production was independently associated with infection of domestic origin and the isolates of foreign origin significantly more often harbored the genes *cj0486 *and *ceuE*, findings also verified with a multivariate analysis.

GGT is an enzyme present in both bacteria and eukaryotes. It has a role in glutathione and glutamine metabolism in *C. jejuni *[30]. The presence of *ggt *has been shown to vary among *C. jejuni *isolates [20,31] and we recently demonstrated that *ggt *was common among human and chicken *C. jejuni *isolates but significantly less common among bovine isolates [25]. GGT activity in *C. jejuni *has been suggested to affect the persistent colonization of the avian gut [20] and in a mouse model for *C. jejuni *it was shown to enhance colonization [30]. In our study, GGT-production was present in 15% of the isolates and associated with bloody diarrhea in the univariate analysis, although the latter, due to a lack of data available, could not be further analyzed in a multivariate analysis. Interestingly, the domestic *C. jejuni *isolates were able to produce GGT significantly more often than the imported isolates, a finding also verified by the multivariate analysis. PFGE typing of all domestic isolates verified the sporadic nature of the domestically acquired infections confirming also that GGT production was not linked with a certain genotype.

The gene *cj0486*, encoding a putative sugar transporter and suggested to be related to invasiveness [13], as well as the gene *ceuE*, encoding a transport protein for uptake of the siderophore enterochelin [32] were detected in 49% and 86% of the isolates in the present study, respectively, in line with some other reports [19]. Although their presence was not associated with the outcome of the disease, interestingly they were significantly more often found among isolates of foreign than among those of domestic origin. Furthermore, the isolates lacking *ceuE *seemed to cause infections requiring hospital treatment, but this finding was not verified by the multivariate analysis. In addition to the typing of all domestic isolates, PFGE typing of travel-associated isolates from July indicated that almost all patients had unique genotypes and none of the studied characteristics was linked with a genotype. Travel-associated isolates originated from a total of 36 different countries.

Only few studies have been promising in trying to show correlation between putative virulence factors or the characteristics of *C. jejuni *and the outcome of the disease. Of the different *C. jejuni *markers studied in the present report, GGT production and the presence of *cgtB *were associated with bloody stools in the univariate analysis, but the other putative virulence factors did not correlate with any specific clinical findings. The ß-1,3 galactosyltransferase gene *cgtB *in the LOS gene clusters A and B involved in the biosynthesis of ganglioside-like LOS [16,17] also showed a trend of being associated with longer-lasting diarrhea. *C. jejuni *LOS gene clusters A, B and C have even earlier been associated not only with a more severe outcome of the disease as characterized by bloody stools and longer duration of diarrhea but also with the development of post-infectious complications [33]. However, in the present study, the other ß-1,3 galactosyltransferase gene *wlaN*, expressed in the LOS gene cluster C [34] was not associated with any clinical characteristics. The gene *ciaB *has been suggested to be involved in invasiveness [11,12] and thus, could be needed for the development of the disease. Indeed, all except two of the 166 isolates in our study were *ciaB *positive, which is in line with earlier reports [19,35]. The presence of another putative virulence factor the gene *pldA*, encoding phospholipase A [14], was detected in the present study to a somewhat lower extent (61%) as compared to other reports (91-100%) [19,35], and did not correlate with the clinical outcome of the disease. As CDT and *virB11 *were concerned, our results supported those of others showing CDT activity in the great majority [19] but the presence of *virB11 *in only a tiny proportion [13,19] of clinical *C. jejuni *isolates. Thus, it seems very unlikely that these particular markers would play a role in the diversity of the outcome of the human disease.

## Conclusions

In conclusion, we suggest for the first time that GGT production could be a marker associated with a more severe outcome of *C. jejuni *infection as characterized by bloody stools, however, additional work is needed to clarify the importance of this finding. Furthermore, to the best of our knowledge, this is the first report to describe the presence of putative virulence markers significantly and independently to differ between *C. jejuni *isolates of foreign and domestic origin. Whether this also reflects the different sources of *C. jejuni *infections locally in Finland remains to be studied.

## Competing interests

The authors declare that they have no competing interests.

## Authors' contributions

BF participated in the design of the study, collected and analyzed data, performed statistical analyses and prepared the draft manuscript. PE participated in the selection of virulence factors, performed and supervised some PCR experiments. HH performed the PFGE and conducted some PCR experiments. SS provided expertise in statistical analyses. MLH participated in the design of the study and supervised the performance of some experiments. HR participated in the design of the project, coordinated and supervised the study and helped to draft the manuscript. All authors provided ideas and comments on the draft manuscript and approved the final manuscript.
